# A Rare Case of DRESS (Drug Reaction with Eosinophilia and Systemic Symptoms) Syndrome with Cholecystitis in a Patient on Levetiracetam

**DOI:** 10.7759/cureus.4245

**Published:** 2019-03-13

**Authors:** Tushi Singh, Masooma Niazi, Kishore Karri, Donald Rudikoff, Efrain Gonzalez

**Affiliations:** 1 Internal Medicine, Bronx Lebanon Hospital Icahn School of Medicine at Mount Sinai, New York, USA; 2 Pathology, Bronx Lebanon, New York, USA; 3 Internal Medicine, University of Kentucky, Lexington, USA; 4 Dermatology, Bronx Lebanon, New York, USA; 5 Infectious Disease, Bronx Lebanon, New York, USA

**Keywords:** adverse reaction, cholecystitis, drug rash with eosinophilia and systemic symptoms

## Abstract

Drug reaction with eosinophilia and systemic symptoms (DRESS) syndrome is a rare idiosyncratic drug reaction with a mortality of up to 10%. As the name suggests, it is characterized by skin rash, eosinophilia, and systemic symptoms resulting from the involvement of visceral organs. We present a case of DRESS in a patient who was on both lamotrigine and levetiracetam, where levetiracetam turned out to be the inciting agent. The interesting features of the case include the onset of symptoms about 70 days after the initiation of levetiracetam, the lack of prominent eosinophilia and the involvement of the gall bladder, which was previously unknown with Levetiracetam. It also reinforces the importance of using the RegiSCAR score in the diagnosis of DRESS. The symptoms resolved over the next few months after drug withdrawal.

## Introduction

Drug reaction with eosinophilia and systemic symptoms (DRESS) syndrome is a rare but potentially life-threatening condition with a mortality rate of 10% [1]. Levetiracetam is a widely prescribed antiepileptic known for its favorable side-effect profile. While DRESS is more common with other anticonvulsants, levetiracetam is a rare cause of DRESS. Cholecystitis in levetiracetam-induced DRESS has not been reported yet, to the best of our knowledge. We present a case with a 70-day lag in the manifestation of DRESS after initiating levetiracetam. The diagnostic challenge was posed by the fact that the patient was on both levetiracetam as well as lamotrigine.

## Case presentation

A 36-year-old female was discovered to have an astrocytoma after she presented with new onset seizures. She was started on levetiracetam to control the seizures. Seven weeks later, she presented with break-through seizures while the serum levetiracetam level was therapeutic. Lamotrigine was then added to her medications. Four weeks later, she presented with a rash, right upper quadrant abdominal pain, and fever for four days. She denied any nausea, vomiting, diarrhea, or joint pains. Her medical history was significant for allergies to nuts and pollen. The family history was not significant. She denied having traveled out of the city in the last 18 months. She was a stay-at-home mother of one child.

**Figure 1:**
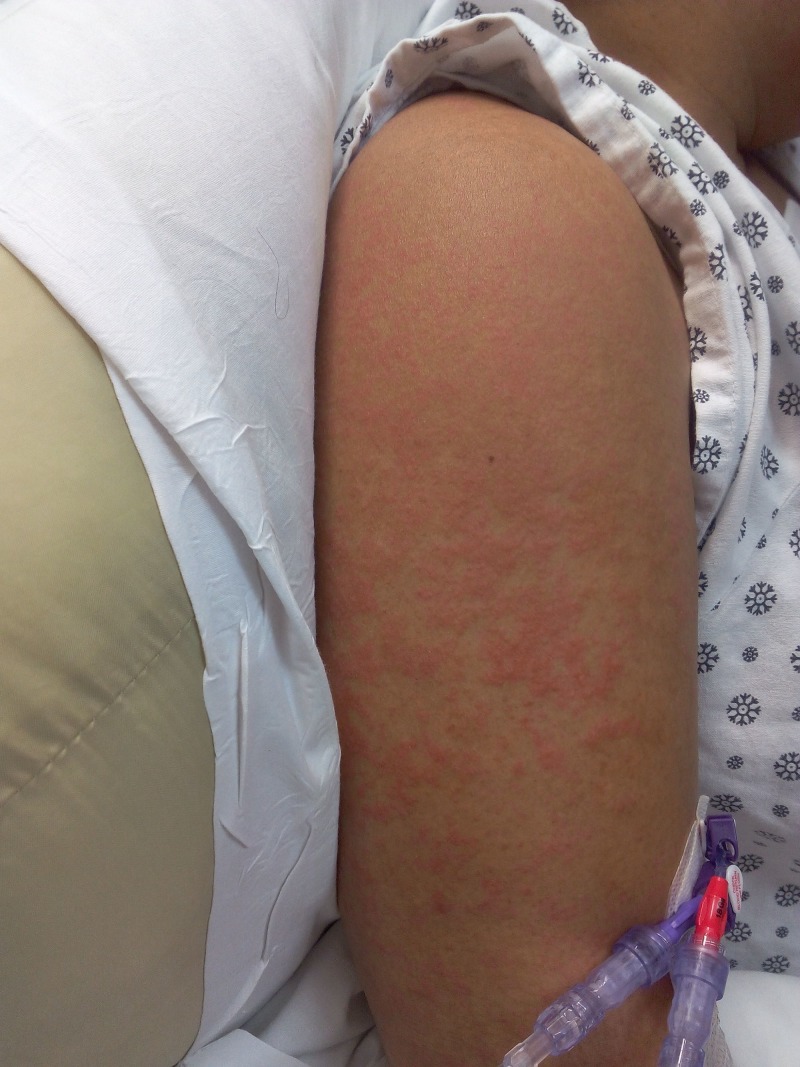
Reticulated erythematous rash on the upper limb on presentation

**Figure 2:**
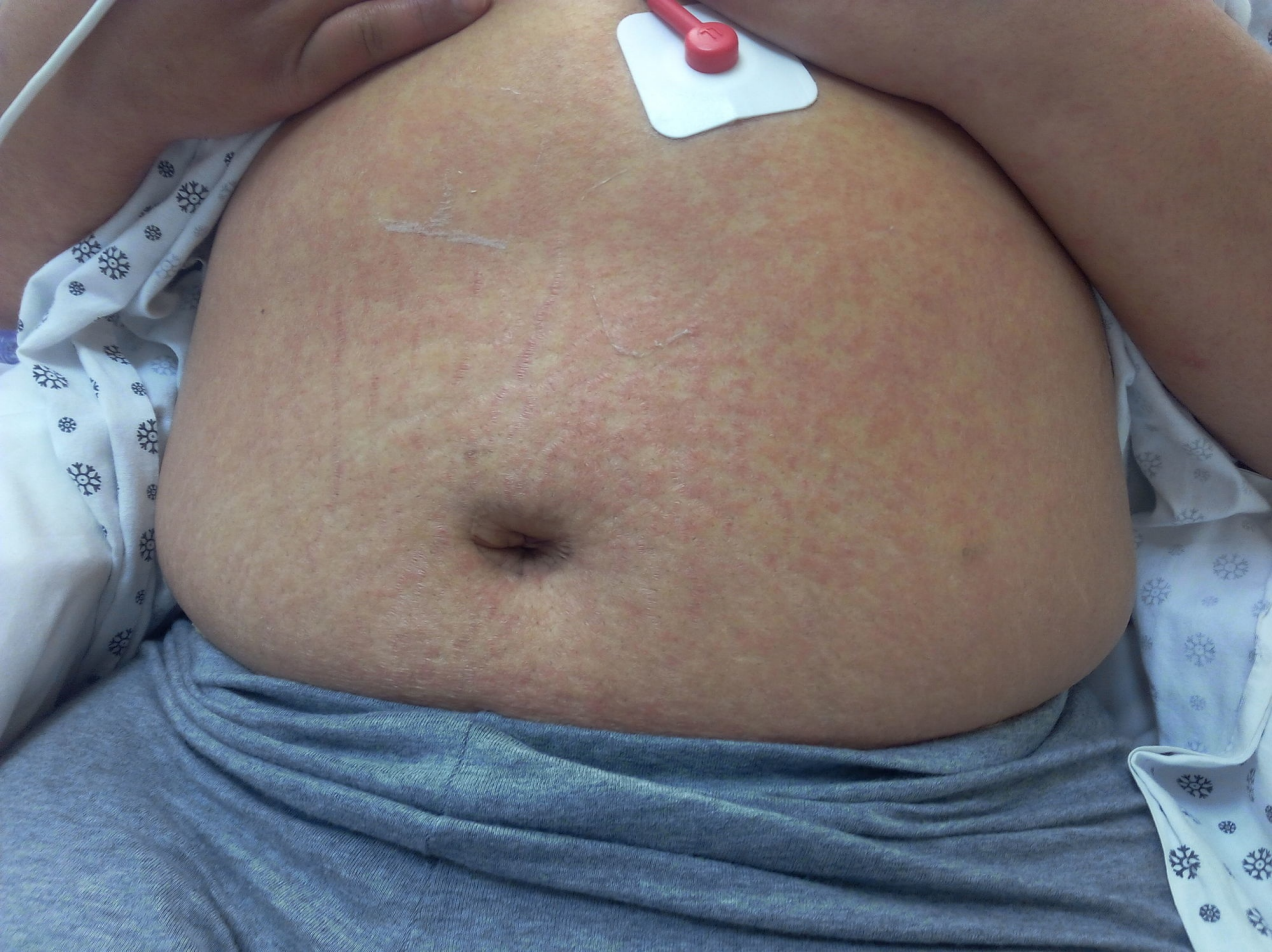
Reticulated erythematous rash on the trunk on presentation

**Figure 3:**
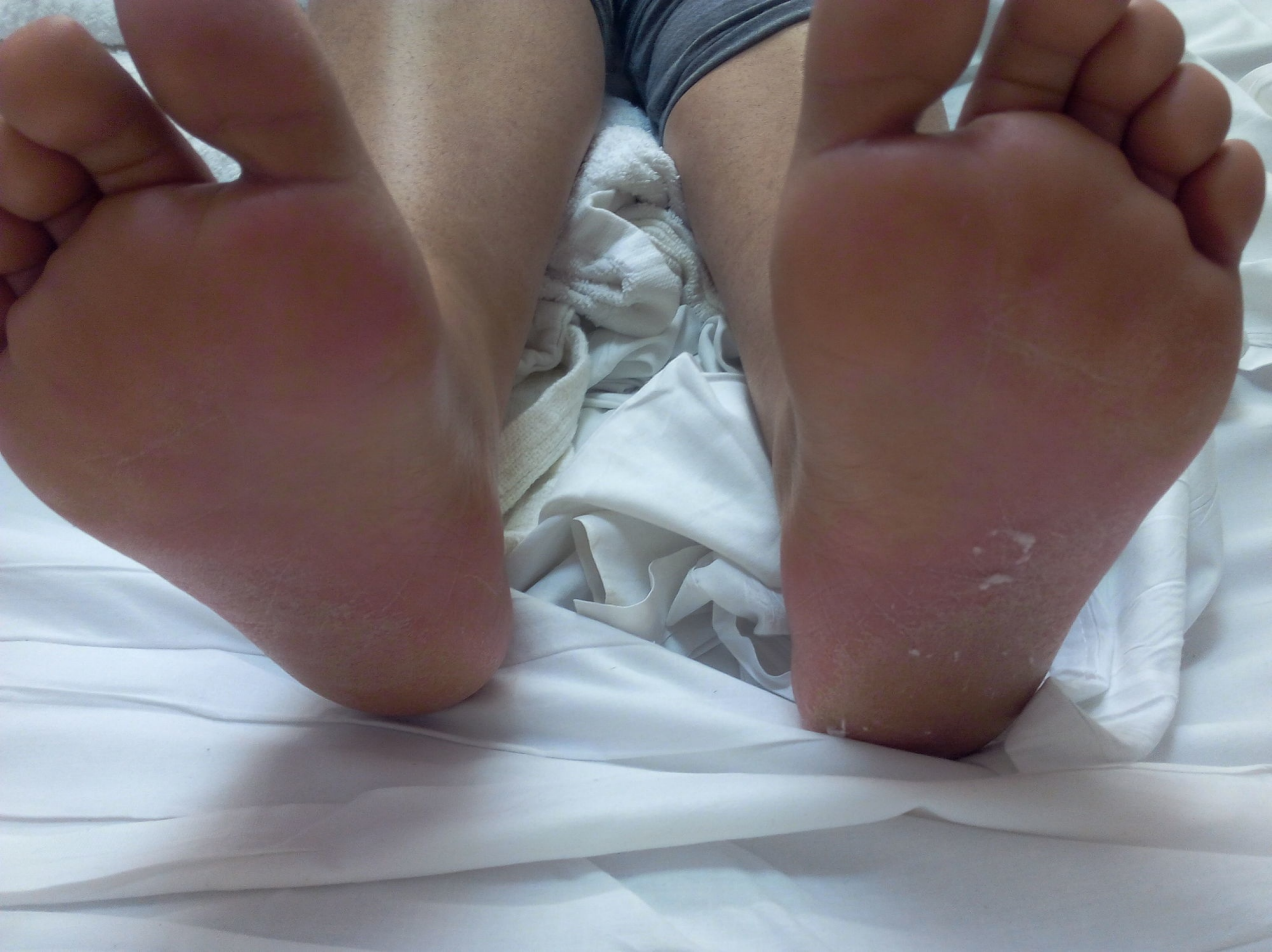
Swelling and rash on the soles: Day 2

**Figure 4:**
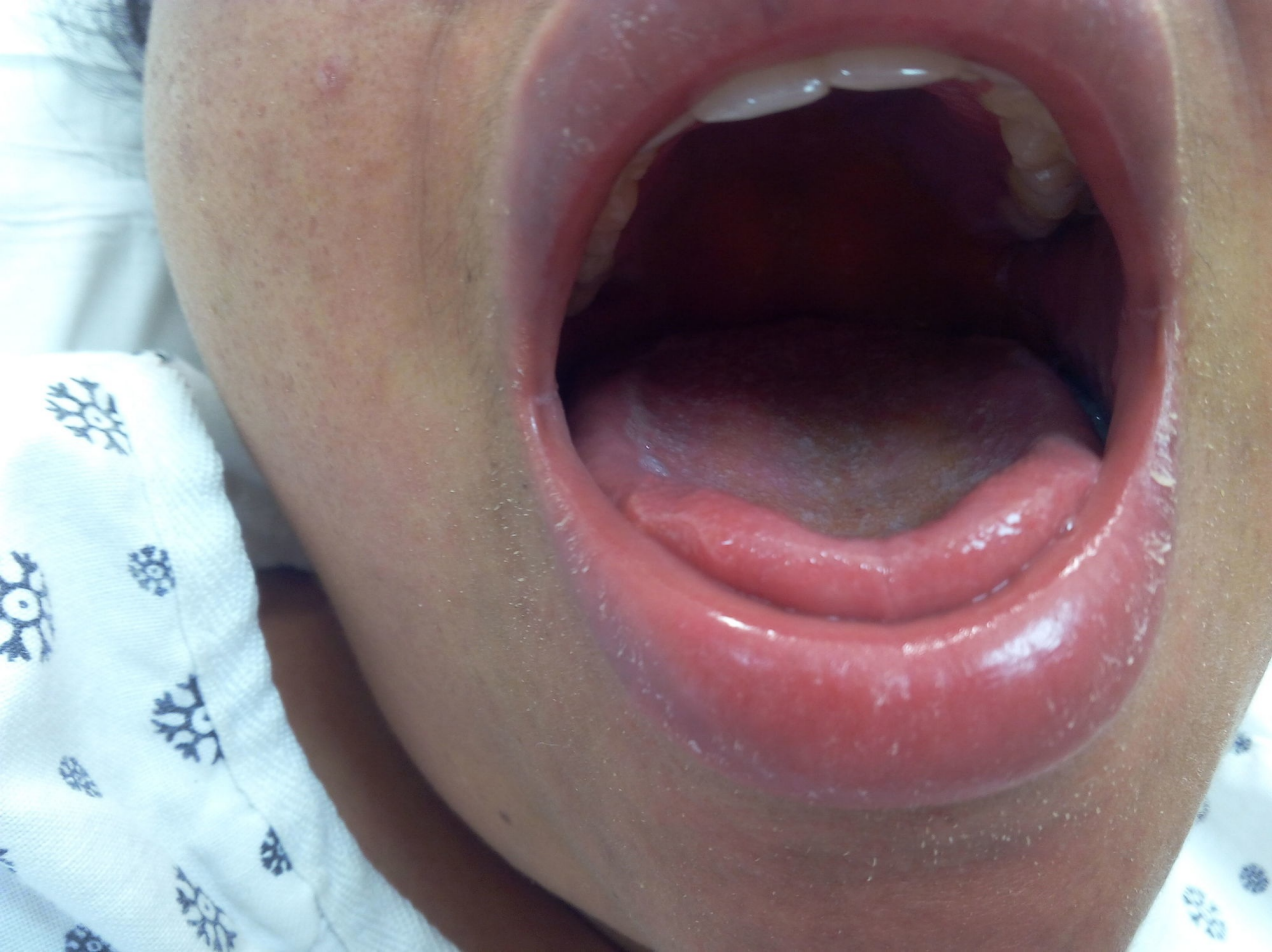
Facial edema and lip swelling: Day 2

**Figure 5:**
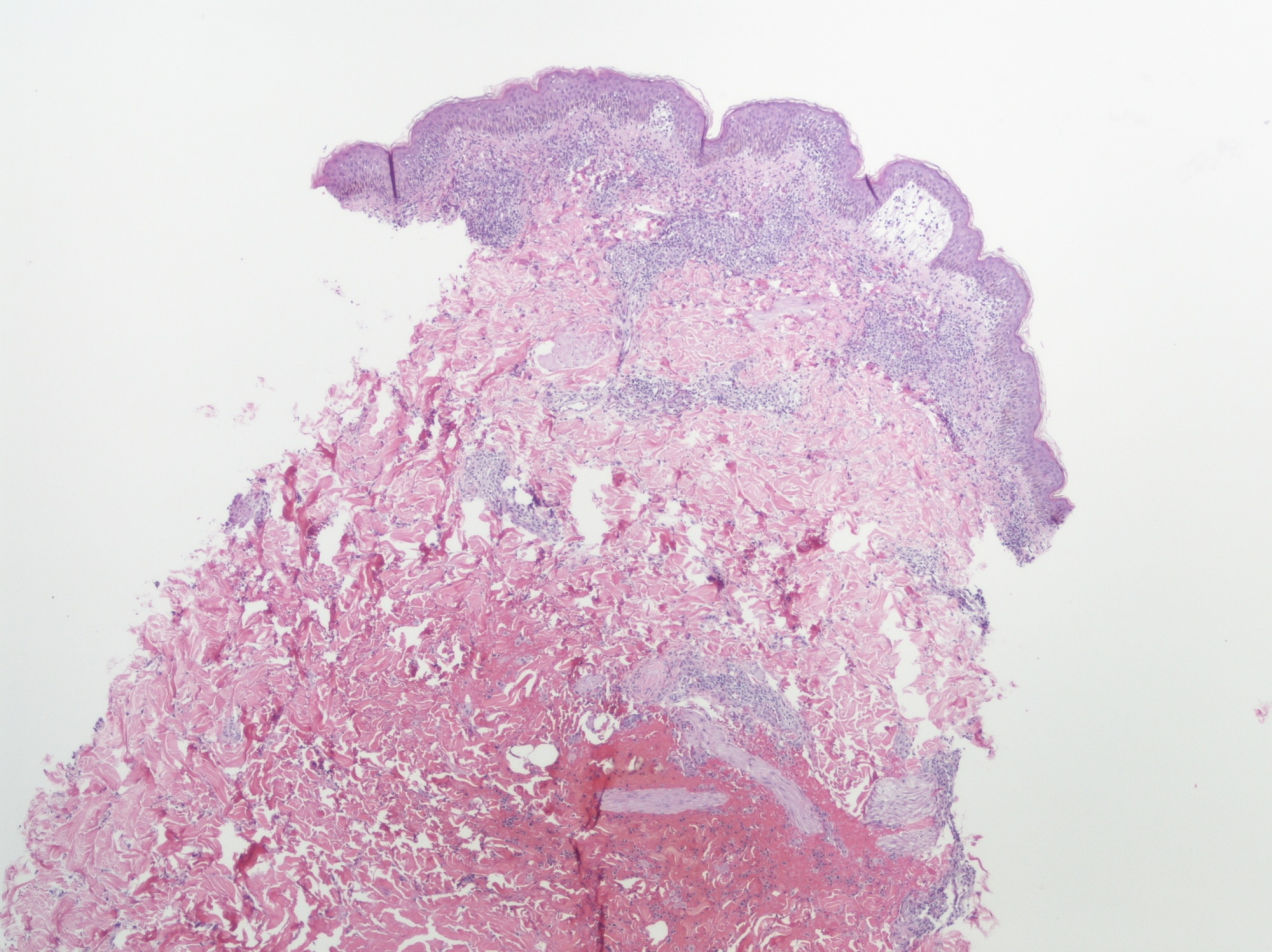
Skin biopsy on low magnification Biopsy with epidermis, dermis, and subcutaneous tissue showing superficial and mid-dermal perivascular dermatitis consistent with a hypersensitivity reaction.

**Figure 6:**
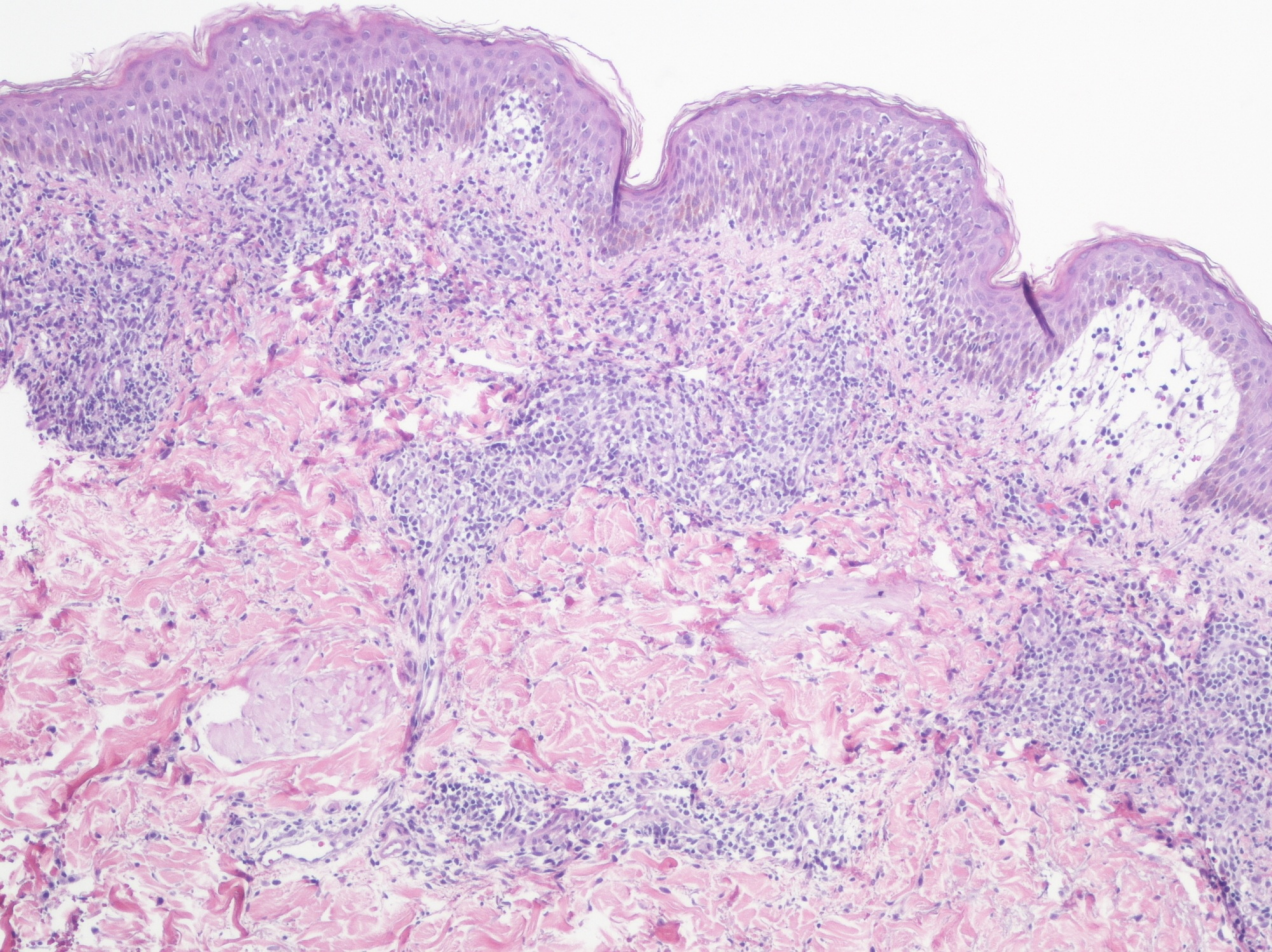
Skin biopsy on high magnification (x100) Biopsy showing perivascular infiltrate consisting of lymphocytes, macrophages, and rare eosinophils.

On physical examination, she was in mild distress due to abdominal pain, febrile to 38 degree Celsius, pulse of 110 per minute, respiratory rate of 18 breaths per min, blood pressure of 124/77 mm Hg in the right upper limb in the supine position, and oxygen saturation of 98% on room air. A reticulated macular erythematous rash was noted on the upper extremities (Figure 1) and trunk (Figure 2). The head and neck exam did not demonstrate any localized swelling, lymphadenopathy, or icterus. There was no oral ulceration or conjunctival injection. Cardiac and lung exam were within normal limits. The abdominal exam revealed right upper quadrant tenderness with Murphy’s sign. Initial lab data demonstrated an eosinophil count of 4 x 10^5/ml, elevated white cell count, alanine aminotransferase (ALT) of 1231 units/L, and aspartate aminotransferase (AST) of 1026 units/L. Alkaline phosphatase was 362 units/L. Lamotrigine was stopped as DRESS was considered. Over the next 48 hours. there was no improvement, against expectations. An ultrasound of the abdomen followed by magnetic resonance cholangiopancreatography confirmed the presence of acalculous cholecystitis. Antibiotics were administered for cholecystitis. Two days later, her abdominal pain subsided but she remained febrile (T-max of 39.3 Deg C). By now, the reticulated erythema spread to her face and lower extremities (Figure 3). Her face became edematous, and she developed enlarged salivary glands and lower lip edema (Figure 4). Her pharynx was noted to be erythematous and her tonsils were enlarged and covered with exudates. Cervical and submandibular lymphadenopathy developed as well. At this time, an alternate diagnosis was entertained while she did not improve over the next one week. She was immune to hepatitis A and B. Antibody to hepatitis C was negative. The antinuclear antibody, anti-smooth muscle antibody, antimitochondrial antibody, and liver-kidney microsomal assay were negative. Serum ceruloplasmin was within normal limits. Serum toxicology showed a salicylate and acetaminophen level within normal limits. Urine toxicology was negative for substances of abuse. Serum titers for rubella, rubeola, mumps, Epstein Barr virus, cytomegalovirus, varicella zoster, and human herpesvirus-6 were negative. Anti-streptococcal titers, throat culture for Streptococcus, and diphtheria culture were sent as well and reported no growth or negative growth. The cell count peripheral smear did not demonstrate mononucleosis or atypical lymphocytosis. A chest radiograph demonstrated a newly developed right-sided pleural effusion without evidence of consolidation. A skin biopsy was done, which confirmed perivascular dermatitis such as that seen in drug hypersensitivity (Figures 5-6).

About 10 days after presentation, the levetiracetam was discontinued because of the lack of improvement in symptoms with the cessation of lamotrigine. With that, she began to improve gradually with the disappearance of the skin rash over the next few days, resolution of the facial and the lymph node swelling, as well as a return of transaminases to the baseline over the next few weeks.

## Discussion

Drug reaction with eosinophilia and systemic symptoms (DRESS) syndrome is a potentially life-threatening hypersensitivity reaction first described with phenytoin in the 1940s [2]. Anticonvulsants and sulfonamides are the most frequently implicated drugs [3]. DRESS usually occurs two to eight weeks after exposure to the trigger medication, and it is characterized by fever, rash, lymphadenopathy, and facial swelling. Cell counts usually demonstrate eosinophilia and leukocytosis. Inflammation of the visceral organs, namely, the liver, kidney, heart, and skin, is also a hallmark of the disease. [4] The pathogenesis is not completely understood, and a combination of genetic deficiencies in drug metabolizing enzymes [5] and the reactivation of the herpes virus due to interaction with the drug [6] has been implicated. The RegiSCAR score (Table 1) is a widely used scoring system that assists in the diagnosis of DRESS where a score of 5 and above confirms the diagnosis [7]. The scoring system uses the clinical, serological, as well as histological aspects of the condition to assign scores. It is broken down as shown below. Our patient met the criteria for DRESS with a score of 6. The scoring points included enlarged lymph nodes (cervical and submandibular) (+1) and skin rash covering over 50% of the body surface area (+1) with edema and scaling (+1), visceral organ involvement (lungs, liver, and gallbladder) (+2). Viral infections, including hepatitis A, B, C and measles, rubella, mumps, as well as ANA, mycoplasma, chlamydiae, and blood cultures were negative (+1). 

**Table 1:** RegiSCAR scoring system Final score <2 - Not DRESS, 2-3 - Possible, 4-5 - Probable, >5 - Definite DRESS: drug reaction with eosinophilia and systemic symptom syndrome; BSA: body surface area

Features	No	Yes	Unknown
Fever, >38.5deg C	-1	0	-1
Enlarged lymph node, >1cm	0	1	0
Eosinophilia >700 or >10% / >1500 or >20%	0	1 / 2	0
Atypical Lymphocytes	0	1	0
Skin Rash >50% BSA	0	1	0
At least two of edema, infiltration, purpura or scaling	-1	1	0
Biopsy suggesting DRESS	-1	0	0
Internal organ involvement 1, 2 or more	1	2	0
Resolution in over 15 days	-1		-1
At least three biological investigations done to rule out an alternative diagnosis	0	1	0

It has been shown that DRESS may be associated with acalculous cholecystitis [8] but reports remain incidental and too few to arrive at a prediction. Only five cases of levetiracetam-induced DRESS have been reported so far [9-11]. Only three cases of cholecystitis associated with DRESS have been reported [12-13]. Allopurinol has been the implicated drug in these cases. To the best of our knowledge, this is the first reported case of levetiracetam-induced DRESS that is associated with cholecystitis. Our case stands out for the prolonged latency of over 70 days instead of the recorded average of one to eight weeks [14] from drug initiation to symptom onset. The lack of eosinophilia on presentation and the presence of lamotrigine in the medication list threw us off the mark but the gradual development of her symptoms even a week after withdrawal of lamotrigine helped us zero in on the inciting drug. This was further aided by the initiation of resolution within 24 hours of withdrawal of levetiracetam. Usually, the resolution occurs over about 6-9 weeks [15]. Our patient had a complete clinical recovery in about eight weeks but the liver functions took over 12 months to resolve. Steroids have been used for the treatment of DRESS in the past but its use remains controversial. As our patient improved in line with our expectations after cessation of levetiracetam, we decided to adopt a wait-and-watch policy in keeping with the patient's wishes. She was started on topiramate for seizure control and has remained well on it.

## Conclusions

Although rare, levetiracetam has been established to cause DRESS. It is important for physicians to entertain the diagnosis when the patient meets the criteria even if the clinical hallmarks (eosinophilia, for example) are absent. DRESS is also associated with acalculous cholecystitis. Early discontinuation of the offending medication leads to quicker recovery.
